# Phytochemical *S*-methyl-L-cysteine sulfoxide from Brassicaceae: a key to health or a poison for bees?

**DOI:** 10.1098/rsob.240219

**Published:** 2024-12-11

**Authors:** Saetbyeol Lee, Pavel Dobes, Jacek Marciniak, Anna Mascellani Bergo, Martin Kamler, Petr Marsik, Radek Pohl, Dalibor Titera, Pavel Hyrsl, Jaroslav Havlik

**Affiliations:** ^1^ Department of Food Science, Faculty of Agrobiology, Food and Natural Resources, Czech University of Life Sciences Prague, Prague, Czech Republic; ^2^ Department of Experimental Biology, Faculty of Science, Masaryk University, Brno, Czech Republic; ^3^ Bee Research Institute, Maslovice, Czech Republic; ^4^ Institute of Organic Chemistry and Biochemistry of the Czech Academy of Sciences, Prague, Czech Republic

**Keywords:** phytochemical, Brassicaceae, *S*-methyl-L-cysteine sulfoxide, *Apis mellifera*, rapeseed, toxicity

## Abstract

Intensive agricultural practices impact the health and nutrition of pollinators like honey bees (*Apis mellifera*). Rapeseed (*Brassica napus* L.) is widely cultivated, providing diverse nutrients and phytochemicals, including *S*-methyl-L-cysteine sulfoxide (SMCSO). While the nutritional impact of rapeseed on bees is known, SMCSO’s effects remain unexplored. We examined SMCSO and its related metabolites—3-methylthiolactic acid sulfoxide and *N*-acetyl*-S*-methyl-L-cysteine sulfoxide—analysing their seasonal fluctuations, colony variations and distribution in body parts. Our findings showed that these compounds in bee gut vary among colonies, possibly due to the dietary preferences, and are highly concentrated in bodies during the summer. They are distributed differently within bee bodies, with higher concentrations in the abdomens of foragers compared with nurses. Administration of SMCSO in a laboratory setting showed no immediate toxic effects but significantly boosted bees’ antioxidant capacity. Long-term administration decreased bee body weight, particularly in the thorax and head, and altered amino acid metabolism. SMCSO is found in the nectar and pollen of rapeseed flowers and highly accumulates in rapeseed honey compared with other types of honey. This study reveals the dual impact of SMCSO on bee health, providing a basis for further ecological and physiological research to enhance bee health and colony sustainability.

## 1. Introduction

Intensive agricultural practices significantly alter the landscape, posing challenges for pollinators [1]. Monocultures, which are characteristic of intensive farming, are associated with pollinator decline due to factors such as pesticide use, reduced biodiversity and poor nutritional diversity [1]. These large-scale, single-crop systems can be highly attractive to bees due to their high nectar concentrations [2]. However, they often fail to provide sufficient quality pollen, negatively impacting bee development and increasing mortality rates [3,4]. Moreover, monocultures provide resources only during short blooming periods, turning fields into ‘green deserts’ once flowering ends, unable to support pollinator colonies for the entire growing season [5,6]. This lack of continuous resources contributes to the overall decline in pollinator health and population sustainability [1].

A prime example of these challenges can be seen with rapeseed (*Brassica napus* L.) cropping. Rapeseed is extensively cultivated, with intensive monoculture practices covering nearly 15% of arable land in the Czech Republic and approximately 5.5% in the EU [7]. Although rapeseed is considered a self-pollinating plant species [8], insect pollination—especially by the European honey bee (*Apis mellifera* L*.*)—can significantly enhance both the yield and quality of rapeseed [9]. Moreover, rapeseed pollen is highly nutritious for bees and results in high honey production when beehives are placed near flowering fields [10,11]. Brassicaceae plants, including cultivated species like rapeseed and non-cultivated species such as wild radish (*Raphanus raphanistrum* L*.*), garlic mustard (*Alliaria petiolata* (M.Bieb.) Cavara & Grande) and thale cress (*Arabidopsis thaliana* (L.) Heynh.), are primarily known for their high concentrations of glucosinolates, sulfur-containing phytochemicals that deter insect pests [12,13]. There is some evidence that glucosinolates may affect honey bee health, particularly in reducing *Nosema* infections [14,15]. However, the overall impact of these phytochemicals on honey bees and the level of exposure remain unclear.

As generalist pollinators, honey bees encounter a diverse array of phytochemicals while foraging. Phytochemicals present in nectar and pollen can impact the behaviour and health of honey bees and other pollinators, with effects ranging from negative to neutral to positive [16]. Thymol, for instance, exhibits inhibitory effects on parasites in bumblebees and reduces *Nosema* infections in honey bees, suggesting a potential role for this compound in enhancing bee health and resilience against infections [17–19]. However, adverse effects of thymol, such as a decrease in fat body mass and vitellogenin gene expression, have been observed in thymol-fed honey bees [20]. Additionally, while caffeine is repellent to honey bees at higher concentrations [21], a low dose of caffeine in floral nectar has been found to improve the learning and memory of bees [22]. Quercetin, a flavonoid with antioxidant properties found in many plant species [23], can also impact bee behaviour. Honey bee workers fed high concentrations of quercetin initiate ovarian development, exhibit reduced queen care behaviour, and show aggression towards the queen [24].

Rapeseed flowers contain multiple phytochemicals beyond glucosinolates, including gallic acid, kaempferol, catechin and quercetin [23]. However, as a member of the Brassicaceae family, rapeseed also contains high levels of *S*-methyl-L-cysteine sulfoxide (SMCSO), a compound known for its toxicity in ruminant animals, causing anaemia [25]. Interestingly, SMCSO is abundant in cruciferous vegetables, often exceeding glucosinolate contents [26]. Although SMCSO is toxic to ruminant animals, it has been reported to have anti-carcinogenic, anti-diabetic and cardiovascular benefits in rats [27–29].

In a previous ^1^H NMR metabolomics study [30], we described a robust biomarker—an unknown compound characterized by a CH_3_ singlet at 2.83 ppm. This compound was 13 times more abundant in summer bees, which have shorter lifespans in temperate climates, compared with winter bees [31], enabling us to distinguish between the two bee populations. In the current study, we identified this compound as SMCSO, along with its catabolites, 3-methylthiolactic acid sulfoxide (3Me-TLA SO) and *N*-acetyl*-S*-methyl-L-cysteine sulfoxide (NAc-SMCSO). These compounds have been also identified as biomarkers in human studies linked to cruciferous vegetable consumption [32,33]. Their consistent detection during our metabolomic analysis in bees prompted further investigation into their biological roles.

During the rapeseed blooming season, bees are likely to be naturally exposed to SMCSO, especially when their hives are situated near rapeseed fields. Given the critical role of honey bees in pollination and the exposure of bees to rapeseed fields, it is essential to understand how SMCSO affects bee health and metabolism. However, little is known about the distribution of SMCSO among rapeseed cultivars, its distribution and metabolism in bee tissues, or whether it has toxic or beneficial effects on bee health and nutrition, as these aspects have not yet been thoroughly investigated. Some studies have suggested that SMCSO may serve as a defensive metabolite against insect herbivory on rapeseed root and flower [34,35]. In relation to SMCSO and honey bees, one study recently reported that SMCSO is a characteristic marker exceptionally high in honey, bee bread and royal jelly from bees exposed to rapeseed [36]. Despite its potential bioactivity, the impact of SMCSO and its metabolites on honey bees remains largely unexplored. Therefore, this study aims to examine the seasonal fluctuations, distribution across various body parts, disparities between nurses and foragers, and variations between colonies for the three metabolites SMCSO, 3Me-TLA SO and NAc-SMCSO. Additionally, we investigated the metabolomic alterations in both the bodies and guts of honey bees following acute and long-term administration of SMCSO. This includes exploring the toxicity of SMCSO and its impact on antioxidant and antimicrobial defences. We also measured SMCSO concentrations in rapeseed flower nectar and pollen, and then analysed three different types of honey to determine the presence of this compound. This research provides a comprehensive investigation into the physiological impacts of SMCSO and its metabolites in honey bees, offering novel insights that may have implications for honey bee health, pollinator conservation and sustainable agricultural practices.

## 2. Results

**Figure 1.**
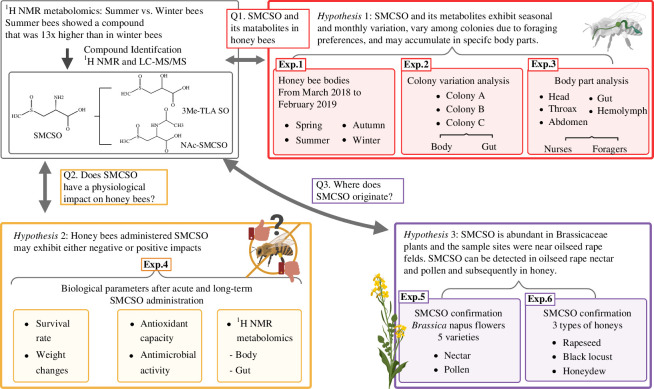
Experimental design and overview of this study. The study addressed three research questions by conducting six experiments on honey bees collected from managed colonies, feeding them experimentally with SMCSO and analysing their food sources and products.

To elucidate the diverse aspects of SMCSO and its metabolites in honey bee physiology and highlight their potential biological roles and environmental presence, we conducted a comprehensive set of experiments (figure 1). We structured our research around three main hypotheses. Firstly, we investigated whether SMCSO and its metabolites exhibit seasonal and monthly variations, differ among colonies due to foraging preferences, and accumulate in specific body parts. Secondly, we examined the impact of SMCSO exposure on honey bee physiology. Lastly, we characterized the environmental presence of SMCSO by detecting it in the nectar and pollen of rapeseed flowers, as well as in multiple types of honey.

### 2.1. Experiment 1: SMCSO, 3Me-TLA SO and NAc-SMCSO concentrations in honey bee bodies change across different months and seasons

**Figure 2.**
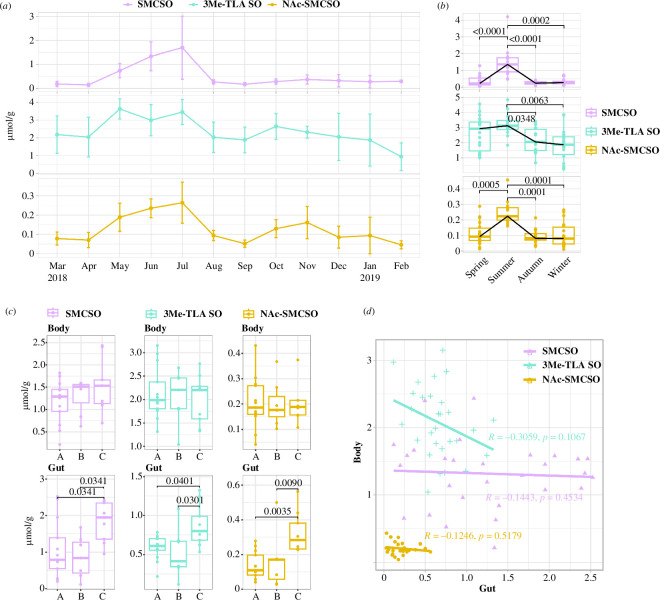
Metabolic variations among colonies in honey bee bodies and guts, as well as monthly and seasonal changes, are observed. (*a*) The monthly fluctuations of SMCSO, 3Me-TLA SO and NAc-SMCSO in honey bee bodies from March 2018 to February 2019 are depicted as mean values with error bars (± s.d.) for the analysed bees (March–November, *n* = 6 each month; December–February, *n* = 4 each month). (*b*) The concentration of SMCSO, 3Me-TLA SO and NAc-SMCSO in the honey bees’ bodies by season. The adjusted *p*-values for statistically significant datasets are displayed (Kruskal–Wallis test; spring, *n* = 18; summer, *n* = 12; autumn, *n* = 18; winter, *n* = 18). (*c*) The concentrations of SMCSO, 3Me-TLA SO and NAc-SMCSO in the body and gut of honey bees were found in three colonies (A, B and C) of a single apiary. The adjusted *p*-values for statistically significant datasets are displayed (Kruskal–Wallis test; A, *n* = 13; B, *n* = 7; C, *n* = 9). (*d*) Metabolite correlations between the body and gut of SMCSO, 3Me-TLA SO and NAc-SMCSO. The body and gut of honey bees (body, *n* = 29; gut, *n* = 29) display a Spearman’s rank-order correlation plot. In the box plots, the line in the middle represents the median, the box represents the first and third quartiles, the whiskers represent the minimum and maximum concentration and the dots depict the individual value. The detailed information can be found in the electronic supplementary material.

To identify monthly and seasonal variations, we monitored the concentrations of SMCSO, 3Me-TLA SO and NAc-SMCSO every month throughout the year. As a first step, SMCSO was identified in a purified extract from honey bee bodies using ^1^H NMR and LC-MS/MS, and the related metabolites 3Me-TLA SO and NAc-SMCSO were identified according to literature research [32,33]. Detailed information regarding the compound annotation is provided in §5 and electronic supplementary material, figures S1–S3. All three metabolites showed a general upward trend from April to July, followed by a downward trend in August and a slight increase from September to October (figure 2*a*). Throughout the entire year, 3Me-TLA SO showed the highest abundance among the three metabolites. The concentrations of SMCSO exhibited a notable increase over the summer months, while in the remaining months the concentration was low (figure 2*a*).

To achieve a broader and more comprehensive analysis, we combined the months into four seasons: spring (March, April and May), summer (June and July), autumn (August, September and October) and winter (November, December, January and February) based on previous research [37,38], local beekeeping practices and foraging activities. The analysis of seasonal variations revealed that the summer season has the highest concentrations of all three metabolites, by contrast to other seasons (figure 2*b*). Consistent with our previous study [30], we observed significantly higher concentrations of SMCSO during the summer compared with other seasons. The concentrations of SMCSO in other seasons were almost negligible, with summer levels being approximately 5 to 6.2 times greater (spring, 0.22 µmol g^−1^; summer, 1.36 µmol g^−1^; autumn, 0.24 µmol g^−1^; winter, 0.27 µmol g^−1^; Kruskal–Wallis test, all adjusted *p *< 0.0002; figure 2*b*; electronic supplementary material, table S1). Concentrations of 3Me-TLA SO, one of the SMCSO metabolites in honey bees, increased significantly during the summer months, approximately 1.2 to 1.7 times higher than in the autumn and winter. However, no significant differences were found when compared with spring (spring, 2.93 µmol g^−1^; summer, 3.13 µmol g^−1^; autumn, 2.05 µmol g^−1^; winter, 1.87 µmol g^−1^; Kruskal–Wallis test, summer–autumn, adjusted *p* = 0.0348; summer–winter, adjusted *p* = 0.0063; figure 2*b*; electronic supplementary material, table S1). Similarly, the concentrations of NAc-SMCSO followed a trend comparable with SMCSO, with a considerable increase of approximately 2.4 to 2.6 times during the summer compared with the other seasons (spring, 0.09 µmol g^−1^; summer, 0.22 µmol g^−1^; autumn, 0.08 µmol g^−1^; winter, 0.08 µmol g^−1^; Kruskal–Wallis test, all adjusted *p *< 0.0006; figure 2*b*; electronic supplementary material, table S1). These findings imply that, by contrast to other seasons, SMCSO and its metabolites reach their peak during the summer, especially in July.

### 2.2. Experiment 2: concentrations of SMCSO, 3Me-TLA SO and NAc-SMCSO in honey bee guts vary among colonies

We conducted a comparative analysis on the concentrations of SMCSO, 3Me-TLA SO and NAc-SMCSO within the bodies and guts of honey bees collected during the summer (June 2017) from three colonies in the same apiary. The results showed no significant differences in the concentrations of SMCSO, 3Me-TLA SO and NAc-SMCSO in the honey bee bodies collected in summer from different colonies, and notable variations were observed among individual bees (figure 2*c*). While bees from colony C had much higher amounts of all three metabolites in their guts than bees from the other two colonies (figure 2*c*; electronic supplementary material, table S2), as confirmed by the Kruskal–Wallis test (all adjusted *p *< 0.05; figure 2*c*). A subsequent analysis investigating the link between body and gut concentrations found no significant correlations (figure 2*d*). The findings indicate that all three metabolite concentrations in the gut vary among colonies, and there is no correlation between gut and body metabolite concentrations.

### 2.3. Experiment 3: nurses and foragers have different body part allocations for SMCSO, 3Me-TLA SO and NAc-SMCSO

**Figure 3.**
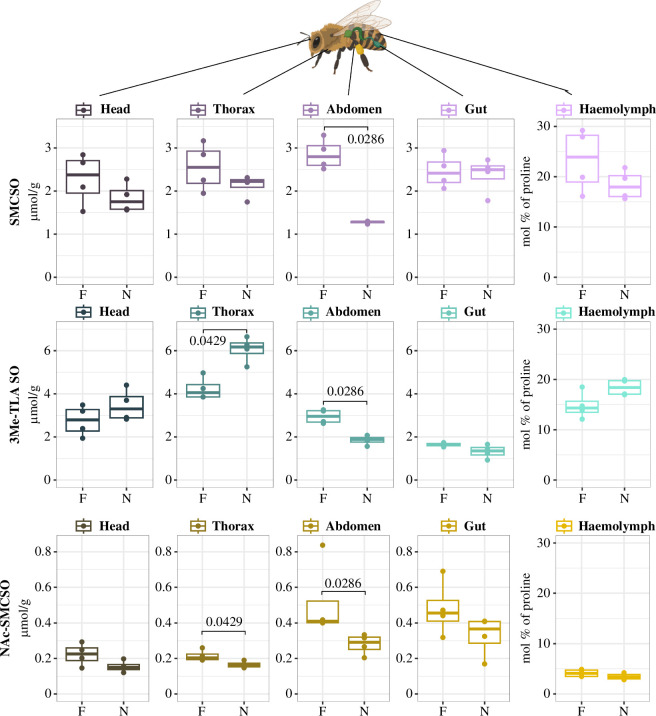
Concentrations of SMCSO, 3Me-TLA SO and NAc-SMCSO in body parts (head, thorax, abdomen, gut and haemolymph) of honey bee nurses and foragers. The line in the middle represents the median, the box represents the first and third quartiles, the whiskers represent the minimum and maximum concentration, and the dots depict the individual value. The adjusted *p*-values for statistically significant results are displayed (Wilcoxon rank-sum test with a Benjamini–Hochberg procedure for false discovery rate correction; F = foragers, *n* = 4; N = nurses, *n* = 4). The detailed information can be found in the electronic supplementary material.

To assess the body part allocation of SMCSO and its metabolites, we examined the distribution and accumulation of metabolites in each body part (head, thorax, abdomen, gut and haemolymph) of worker bees, including foragers and nurses, from a single colony in the summer (July 2020). The concentrations of SMCSO were found to be evenly distributed across all body segments, with higher concentrations observed in foragers. In the abdomen, a clear distinction between the two groups was observed, with foragers having twice the concentration compared with nurses (foragers, 2.8 µmol g^−1^; nurses, 1.28 µmol g^−1^; Wilcoxon rank-sum test, adjusted *p* = 0.0286; figure 3; electronic supplementary material, table S3). Regarding 3Me-TLA SO and NAc-SMCSO, it was observed that the distribution of metabolite concentrations varied among the body segments. The thorax exhibited the highest concentrations of 3Me-TLA SO among all body parts. Specifically, the concentrations in the thorax of nurses were approximately 1.5 times greater than those in foragers (foragers, 4.05 µmol g^−1^; nurse, 6.17 µmol g^−1^; Wilcoxon rank-sum test, adjusted *p* = 0.0429; figure 3; electronic supplementary material, table S3). Additionally, the concentration of 3Me-TLA SO in the abdomens of foragers and nurses differed by approximately 1.6 times (foragers, 2.96 µmol g^−1^; nurses, 1.88 µmol g^−1^; Wilcoxon rank-sum test, adjusted *p* = 0.0286; figure 3; electronic supplementary material, table S3). The concentration of NAc-SMCSO was generally higher in foragers compared with nurses across all body parts. Notably, the thorax concentrations in foragers were significantly higher than in nurses (foragers, 0.2 µmol g^−1^; nurses, 0.16 µmol g^−1^; Wilcoxon rank-sum test, adjusted *p* = 0.0429; figure 3; electronic supplementary material, table S3). Similarly, the abdomen concentrations in foragers were significantly higher than in nurses (foragers, 0.41 µmol g^−1^; nurses, 0.29 µmol g^−1^; Wilcoxon rank-sum test, adjusted *p* = 0.0286; figure 3; electronic supplementary material, table S3). These results show that foragers generally have higher concentrations of all three metabolites than nurses, particularly in their abdomens.

### 2.4. Experiment 4: administration of SMCSO to honey bees did not affect mortality or immune response but resulted in changes in body weight, antioxidant activity and metabolic patterns

To assess whether honey bees benefit from relevant concentrations of SMCSO in nectar or pollen, we conducted acute (AC) and long-term (LT) administration experiments. The AC group received a one-time 1 mg of SMCSO in 1 ml of sucrose solution, while the control group received only sucrose solution. The LT group received 1 mg of SMCSO in sucrose solution ad libitum over the 10 day experiment.

**Figure 4.**
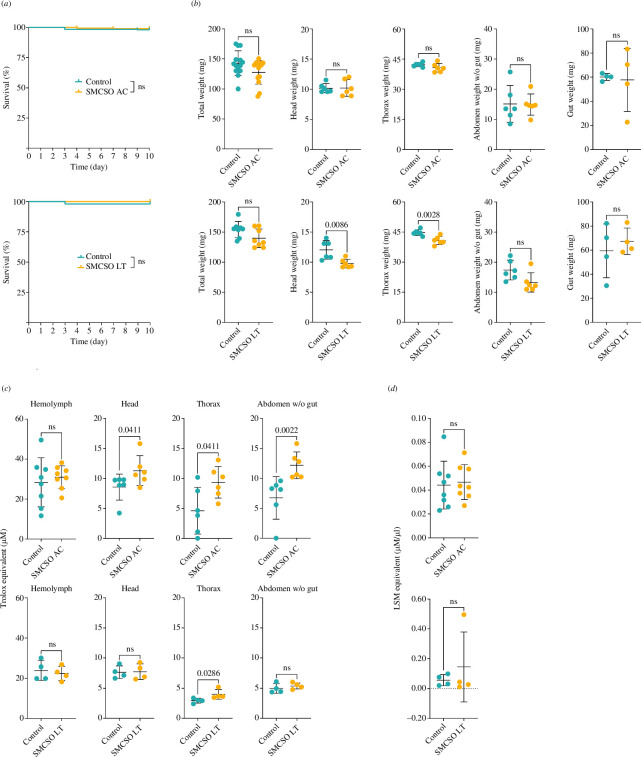
Evaluation of biological parameters after SMCSO administration. (*a*) The survival of newly emerged honey bees fed with SMCSO. Honey bees were exposed to one dose of SMCSO (acute administration, AC; *n* = 100 per each group) and to constant feeding with SMCSO (long-term administration, LT; *n* = 50 per each group), and their survival was scored daily for 10 days. The Mantel–Cox test was used to compare the survival of the control and SMCSO-administered groups; ns = not significant. (*b*) The effect of SMCSO administration on honey bee weight. From left, total weight, weight of head, weight of thorax, weight of abdomen without gastrointestinal tract and venom sac and weight of gut were measured in control bees and bees after acute (AC) or long-term (LT) administration of SMCSO. Data are presented as the mean ± s.d.; *n* = 4–14. Significant differences are indicated above the datasets; ns = not significant (unpaired *t*‐test). (*c*) Antioxidant capacity is expressed as the concentration of standard antioxidant Trolox in haemolymph, head, thorax and abdomen without gastrointestinal tract and venom sac of control honey bees and bees after acute (AC) or long-term (LT) exposure to SMCSO. Data are presented as the mean ± s.d.; *n* = 4–8. Significant differences are indicated; ns = not significant (unpaired *t*‐test). (*d*) Antimicrobial activity is expressed as the concentration of lysozyme (LSM) in the haemolymph of honey bees exposed to acute administration (AC) of SMCSO and long-term (LT) administration of SMCSO. Data are presented as the mean ± s.d.; *n* = 4–8. Significant differences are indicated above the datasets; ns = not significant (unpaired *t*‐test). The detailed information can be found in the electronic supplementary material.

To assess potential toxic effects, we examined the survival rate of honey bees exposed to SMCSO over different AC and LT feeding periods. Compared with the control bees, neither AC nor LT group bees exhibited any significant disparities in survival rate (figure 4*a*). We then assessed the potential impact of SMCSO on weight. We found no statistically significant differences in total weight or individual body part measurements between control and AC bees (figure 4*b*). However, we observed that LT bees tended to have a lower body weight than control bees after LT exposure. Specifically, the head weight of the LT bees showed a decrease (control, 12.03 ± 1.55 mg; LT, 9.77 ± 0.72 mg; electronic supplementary material, table S4) and thorax weight (control, 44.73 ± 1.40 mg; LT, 40.78 ± 2.02 mg; electronic supplementary material, table S4) were 1.2 and 1.1 times lower than the control bees (unpaired *t*‐test, head, *p* = 0.0086; thorax, *p* = 0.0028; figure 4*b*).

The antioxidant capacity measured by the oxygen radical absorption capacity assay has shown that AC bees have increased antioxidant capacities in all analysed body parts, except for the haemolymph, in comparison with control bees (figure 4*c*). Particularly, there was a significant increase in antioxidant capacity observed in the head, thorax and abdomen without a gut (unpaired *t*‐test, head, *p* = 0.0411; thorax, *p* = 0.0411; abdomen without gut, *p* = 0.0022; figure 4*c*). The AC bees’ antioxidant capacity in heads was found to be 1.3 times higher than that of control bees (AC heads, 11.27 ± 2.50 μM; control heads, 8.55 ± 2.16 μM; figure 4*c*; electronic supplementary material, table S5). The thorax and abdomen antioxidant capacities were 2 times and 1.8 times higher in AC bees than in control bees, respectively (AC thorax, 9.34 ± 2.63 μM; AC abdomen, 12.17 ± 2.23 μM; control thorax, 4.60 ± 3.90 μM; control abdomen, 6.73 ± 3.56 μM; figure 4*c*; electronic supplementary material, table S5). LT bees had an increased antioxidant capacity in the thorax, but not in other body parts. In the thorax, LT bees have shown about 1.4 times higher antioxidant capacity than the control bees (LT thorax, 3.99 ± 0.82 μM; control thorax, 2.94 ± 0.40 μM; unpaired *t*‐test, *p* = 0.0286; figure 4*c*; electronic supplementary material, table S5). The SMCSO administration did not have any notable effect on the antimicrobial activity seen in the haemolymph of honey bees. The statistical analysis revealed no significant difference in the antimicrobial activity of the AC and LT bees compared with the control bees (figure 4*d*).

We applied semi-targeted ^1^H NMR metabolomics to analyse honey bee bodies and guts to determine whether the AC and LT administration of SMCSO influenced the metabolite changes in the bodies and guts of caged bees. A total of 77 NMR-identified body and gut metabolites belonged to broad categories showing active metabolism of carbohydrates, amino acids, carboxylic acids, nucleic acids and nucleotides, and amines. The non-annotated signals were embedded in the analysis as 134 spectral buckets (electronic supplementary material, table S6).

**Figure 5.**
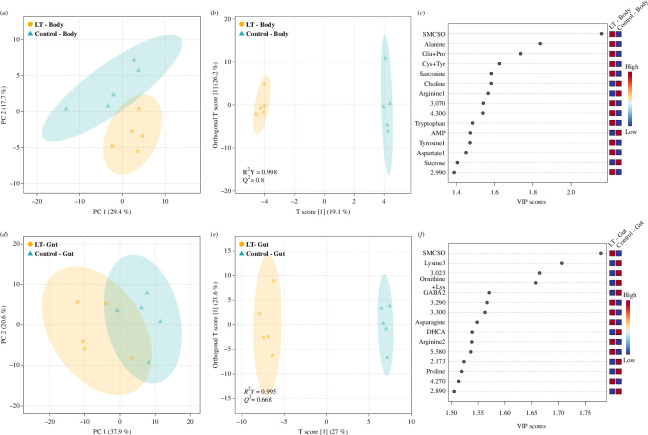
Multivariate analysis of gut and body metabolome of bees following long-term administration of SMCSO. (*a*) PCA score plot and (*b*) OPLS-DA score plot of the body metabolome. (*c*) VIP loading plot from the OPLS-DA of the 15 most contributing metabolites of the body metabolome. (*d*) PCA score plot and (*e*) OPLS-DA score plot of the gut metabolome. (*f*) VIP loading plot from the OPLS-DA of the 15 most contributing metabolites of the gut metabolome. The analyses compare gut or body metabolomes of bees exposed to SMCSO (*n* = 5) with the control group (*n* = 5). LT: long-term administration of SMCSO; Glu+Pro, glutamate and proline-containing bin; Cys+Tyr, cysteine and tyrosine; ornithine+Lys, ornithine and lysine; AMP, adenosine monophosphate; GABA, gamma-aminobutyric acid; DHCA, dihydrocaffeic acid. Unidentified spectral bins are labelled with chemical shift (δ, ppm) values marking the beginning of the interval. A number is appended to the compound name when a single compound is distributed across multiple bins (for a complete list of bins, see electronic supplementary material, table S6).

We then applied multivariate analysis to determine whether the metabolite changes could be used to differentiate between the two groups and identify the key contributing variables. In the principal component analysis (PCA) of the LT administration study, the first two principal components explained 47.1% of the variability between the groups and showed a distinct disparity in the bodies of the control and LT bees (figure 5*a*). Subsequently, orthogonal partial least squares discriminant analysis (OPLS-DA) and permutation test were employed to validate the model, confirming a reliable separation with Q^2^ = 0.8 and R^2^Y = 0.99 (figure 5*b*). The variable importance in projection (VIP) scores were computed to identify the top 15 features reflecting the metabolites that play a significant role in distinguishing between the two groups. These metabolites include SMCSO, alanine, a combination of glutamate and proline, a combination of cysteine and tyrosine, sarcosine and others (figure 5*c*).

When comparing the guts of control bees and LT bees, the PCA displayed a relatively distinctive separation between the two groups, while the first two principal components explained 58.5% of the variance (figure 5*d*). Additionally, a permutation test showed that the OPLS-DA was also able to distinguish between the two groups, indicating a reliable separation (Q^2^ = 0.67 and R^2^Y = 0.99; figure 5*e*). The 15 metabolites, including SMCSO, lysine, gamma-aminobutyric acid (GABA), and several others, were chosen as significant features that differentiated the two groups (figure 5*f*).

Regarding the groups of the AC administration, the multivariate analysis of the honey bee bodies and guts did not demonstrate any clear distinctive differences between the control and AC bees (electronic supplementary material, figure S4). Visual inspection of both bodies and guts has failed to distinguish the two groups on the PCA, and so did the permutation test after OPLS-DA, indicating a poor discrimination validity value that is below 0.5 (AC body, Q^2^ = 0.09 and R^2^Y = 0.95; AC gut, Q^2^ = 0.04 and R^2^Y = 0.99; electronic supplementary material, figure S4). Although SMCSO, 3Me-TLA SO and NAc-SMCSO were detected in both the body and gut of honeybees (electronic supplementary material, table S6), 3Me-TLA SO and NAc-SMCSO did not significantly contribute to the differences between control and treatment groups (AC and LT) based on VIP score analysis (figure 5*c*,*f*; electronic supplementary material, figure S4). Based on these overall findings, we can conclude that SMCSO administration is not toxic to honey bees. Instead, it increases the antioxidant capacity of various body parts. However, it does not have antimicrobial activity and decreases body weight. Additionally, long-term SMCSO administration results in high concentrations of SMCSO in the body and gut and leads to changes in amino acid profiles.

### 2.5. Experiments 5 and 6: SMCSO presence in rapeseed flower nectar and pollen, honey comparison

**Figure 6.**
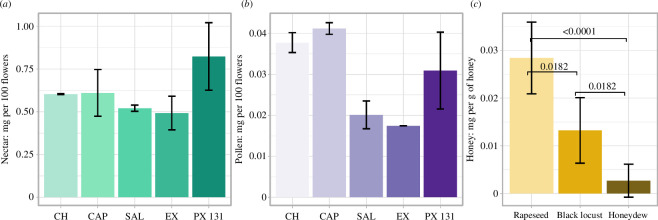
SMCSO is found in the nectar and pollen of rapeseed flowers (*Brassica napus* L.), as well as in honey. (*a,b*) SMCSO concentration in five different varieties of rapeseed flower in (*a*) nectar and (*b*) pollen; data are presented as the mean ± s.d., *n* = 2 for each variety. CH, Chetta; CAP, ES Capelo; SAL, Salute; EX, Exbury; PX 131, PX131. (*c*) SMCSO concentration in three different honeys: rapeseed honey, black locust honey and honeydew honey; data are presented as the mean ± s.d. The adjusted *p*-values for statistically significant datasets are displayed (Kruskal–Wallis test; rapeseed, *n* = 10; black locust, *n* = 10; honeydew, *n* = 10). The detailed information can be found in the electronic supplementary material.

To confirm the presence of SMCSO in rapeseed flowers and honeys, we analysed five different varieties of rapeseed flowers from the Czech Republic: Exbury (EX), Chetta (CH), PX 131, ES Capelo (CAP) and Salute (SAL). Additionally, we examined three different types of honey to determine whether SMCSO is present in them (figure 6). The SMCSO concentration in the rapeseed flower nectar and pollen varied by variety. In nectar, PX131 had the highest average concentration at 0.82 mg per 100 flowers, while the other varieties exhibited relatively similar concentrations (figure 6*a*; electronic supplementary material, table S7). In pollen, SMCSO concentrations also varied among the varieties, with CH and CAP showing high average concentrations of 0.03 and 0.04 mg per 100 flowers, respectively (figure 6*a*; electronic supplementary material, table S7). When analysing honey, the SMCSO concentration was found to differ significantly based on the floral sources. Rapeseed honey had the highest SMCSO concentration at 0.028 mg per gram of honey, followed by black locust honey at 0.013 mg g^−1^ and honeydew honey at 0.0027 mg g^−1^, which was below the detection limit of 0.003 mg (Kruskal–Wallis test; rapeseed–black locust, adjusted *p* = 0.0182; rapeseed–honeydew, adjusted *p *< 0.0001; black locust–honeydew, adjusted *p* = 0.0182; figure 6*c*). The results suggest that SMCSO exhibits variation among flower varieties in both nectar and pollen, with higher concentrations in nectar than in pollen. The presence of SMCSO is more abundant in rapeseed honey compared with other types, such as black locust honey and honeydew honey.

## 3. Discussion

When comparing summer and winter bee metabolites, we identified the presence of SMCSO as unique to summer bees [30]. We then elucidated the dynamics of SMCSO, NAc-SMCSO and 3Me-TLA SO throughout the year, as well as their colony variation and distribution among different body parts. Additionally, we evaluated the physiological impacts of SMCSO on honey bee metabolism and health, as well as its presence in rapeseed nectar, pollen and honey.

SMCSO, a non-proteinogenic amino acid, is a naturally occurring sulfur-containing phytochemical commonly found in various plants of the *Allium* genus within the Amaryllidaceae family and Brassicaceae family [25]. It is present in the highest concentrations in Brussels sprouts, followed by cauliflower and Chinese chives [26]. Notably, the dry weight of SMCSO in cruciferous vegetables is approximately 10 times higher than that of all glucosinolates [26]. This compound is released when plant tissues are macerated or degraded by the gut microbiota [26]. SMCSO produces several derivatives via cysteine sulfoxide lyases: *S*-methyl methanethiosulfinate, *S*-methyl methanethiosulfonate, pyruvate, ammonia, sulfate and dimethyl disulfide [25]. Among these, dimethyl disulfide is highly reactive and can trigger a toxic effect, causing severe haemolytic anaemia in ruminant animals [25]. Consequently, SMCSO is referred to as the ‘kale anaemia factor’ [25]. SMCSO, along with 3Me-TLA SO and NAc-SMCSO, have been identified as urinary biomarkers in individuals who consume cruciferous vegetables or a high-fibre diet [32,33,39]. The two metabolites NAc-SMCSO and 3Me-TLA SO were found to be produced during the degradation of SMCSO via *N*-acetylation and deamination, respectively [39–41]. It is widely known that greater consumption of cruciferous and allium vegetables is associated with a lower incidence of cardiovascular diseases [26]. Despite substantial evidence from human and ruminant animal research, the potential contribution of SMCSO and its derivatives to insect research remains largely unexplored [42].

In the Czech Republic, the primary pollen source for honey bees is the Brassicaceae family [43], with rapeseed in particular being the second most sown crop after wheat in the past decade [44]. Given that the experimental sites were in areas surrounded by farmland and forest with well-established rapeseed cultivation, we hypothesized that the source of SMCSO is the nectar or pollen of Brassicaceae plants, particularly rapeseed flowers (*Brassica napus* L.). To test this hypothesis, we analysed the nectar and pollen of five different rapeseed varieties and confirmed the presence of SMCSO, noting its significantly high concentration in rapeseed honey. Besides the universal presence of SMCSO in Brassicaceae plants, its actual content varies by species, variety, growing season and location [45–47]. The variation of SMCSO content among the five different varieties in nectar and pollen is consistent with these factors. Additionally, we found that rapeseed honey contained substantially higher amounts of SMCSO compared with black locust and honeydew honey, with concentrations being 2 to 10 times greater. The measured SMCSO concentration of 0.028 mg g^−1^ of rapeseed honey is consistent with a recent study by Liu *et al*., which reported 0.075 mg g^−1^ of SMCSO in rapeseed royal jelly and 0.04 mg g^−1^ in rapeseed honey [36]. Additionally, in rapeseed flowers, SMCSO is mainly found in the reproductive organ, specifically in the anther tissue, and not in the perianth. This suggests that this phytochemical may have insect-repellent properties and a higher chance for honey bees to encounter it while foraging [34,48].

Upon comparing the concentrations of these three metabolites in the bodies and guts of honey bees from different colonies during the summer, we observed variation that may indicate differences in the short-term foraging behaviour of each colony. This is evidenced by a notable difference in the concentration of all three metabolites in the guts of bees from neighbouring colonies. These differences in gut metabolites may be influenced by the availability and utilization of various floral sources and foraging preferences [11]. However, the similarity in body concentrations across the colonies implies that the long-term foraging patterns are more consistent. Importantly, the lack of correlation between gut and body metabolite concentrations indicates that the accumulation of bodily reserves takes place over a more extended period, possibly reflecting the integration of metabolites from various foraging trips rather than short-term dietary variations.

A similar tendency of fluctuation was observed for these three metabolites over the course of 1 year. Specifically, the pattern that initiates an upward trend in May and reaches its highest peak during midsummer in June and July resembles the honey bee foraging activity patterns [49], peak of anabolic metabolism [50], colony growth and reproduction [51], and the patterns of seasonal fluctuations of the pollen yield observed in European countries [52]. This trend aligns with the rapeseed flowering period [53], indicating that these seasonal fluctuations of the metabolites can be linked to the flowering and growing periods of plants. A study observed that SMCSO concentrations in rapeseed aerial parts gradually increased as the plant grew [54]. This is similar to how SMCSO concentrations in garlic bulbs and leaves change with growth and maturation [55]. The phytochemical profile of honey also reflects the flowering periods of major nectar and pollen sources, as described in another study. For instance, kaempferol, prevalent in sunflowers, steadily increased, peaking during Colorado’s sunflower bloom in the autumn. Similarly, *p*-coumaric acid, abundant in *Melilotus* spp. flowers, rose from spring to summer and declined in the autumn, following the blooming period [56]. Furthermore, the large variation in SMCSO during June and July suggests potential influences from different foraging locations and flower source availability.

The analysis of SMCSO, NAc-SMCSO and 3Me-TLA SO distribution in nurse and forager bees during the summer indicates significantly higher levels of all three metabolites in the abdomen of foragers compared with nurses. Foragers likely accumulate these metabolites through frequent trophallactic interactions, as they are fed more often than nurse bees [57]. Foragers almost exclusively use sugars for flight, requiring additional energy for their trips and consuming a mixture of nectars [58–61]. They can also use stored nectar from their crops to meet immediate energy demands, which increases their exposure to SMCSO [62,63]. Since SMCSO was primarily found in nectar, foragers have a higher exposure and, thus, a greater likelihood of accumulating these metabolites compared with nurse bees. A study administering ^35^S-labelled SMCSO to humans demonstrated that it is well absorbed from the gastrointestinal tract, with urine being the major route of excretion [41]. A nearly complete recovery of radioactivity was achieved after two weeks [41]. In our three experiments (experiments 1, 2 and 3), 3Me-TLA SO appears to be more abundant in honey bee bodies than SMCSO, possibly due to differences in metabolism and excretion patterns compared with humans. However, the metabolic fate and retention period of these metabolites in honeybees remain undetermined, necessitating further study.

Our laboratory experiments revealed that SMCSO does not directly impact honey bee survival during exposure, indicating that SMCSO administration did not exert toxic effects. However, both AC and LT administrations have shown a trend of decreasing body weight, especially in the head and thorax during LT administration. The head contains a considerable mass of hypopharyngeal gland (HPG), a gland that is crucial for young nurse bees in colony feeding and is sensitive to diet and nutritional stress, causing HPG degradation [64]. Simultaneously, thorax mass serves as an indicator of foraging capacity due to the maturation of flight muscle [65], and weight loss may result from nutritional stress [66]. The finding that SMCSO in continuous ad libitum supplementation significantly lowered the weights of heads and thoraces implies a reduction in the size of HPG and the development of thorax muscles. Therefore, these results could imply a negative impact on honey bee health and growth.

SMCSO supplementation has shown a marked increase in antioxidant capacities in honey bee bodies. Although there is no supporting research in insects, the antioxidant properties of SMCSO have been described in rodents, where its administration to diabetic rats lowered the oxidative stress biomarkers [28,67]. Honey bees respond to some dietary challenges with increases in antioxidant capacity, as observed with substances like zinc, spermidine, melatonin and spirulina; some of these also increase the expression of genes related to antioxidant enzyme function [68–71]. The significantly increased antioxidant capacity in various body parts of honey bees consuming SMCSO, in both the short and long term, suggests an ability to cope with oxidative damage from continuous flight [72]. Additionally, it indicates an improved response to stressors such as pesticide exposure or pathogenic infections [70,73]. Although it has been suggested that the major derivatives of SMCSO induced by enzymatic hydrolysis have antimicrobial effects [25], our study did not confirm their role in honey bee antimicrobial activity in the honey bee haemolymph.

We examined the metabolic changes in the bodies and guts of bees, for both the short- and long-term duration of SMCSO administration. In the AC study, no discernible distinction was observed between the control and treated groups in terms of SMCSO content or any other metabolite. By contrast, LT resulted in a highly specific metabolic profile in both guts and bodies, with SMCSO emerging as the most distinguishing biomarker. The observed metabolic alterations suggest an impact on amino acid metabolism and protein synthesis. Lysine, an essential amino acid, was found at lower levels in the guts, while other free amino acids, especially alanine, accumulated in the body tissues. A significant finding was the substantial reduction of GABA in honey bee guts, a neurotransmitter in the bee brain crucial for olfactory learning and memory performance [74]. Furthermore, the group of metabolites, including arginine, ornithine, lysine and GABA, displaying a decrease in the LT bee guts, are all associated with the polyamine pathway. These polyamines are known to be significant in the proboscis extension response mechanism in honey bee brains, an appetitive component of the bees’ feeding behaviour [75], suggesting potential implications for reduced feeding abilities. Combined with the noted impact on the bee body weight and the reduced size of their heads and thoraces, these findings collectively suggest that SMCSO may have a rather negative effect on protein metabolism and nutritional intake.

Our study introduces SMCSO as a seasonal biomarker in summer bees, along with its metabolites, providing a comprehensive analysis of these compounds and their biological effects on honey bees. Additionally, we provide evidence that SMCSO originates from Brassicaceae plants, such as rapeseed flowers. Despite these advancements, it is essential to acknowledge the study’s limitations. Firstly, our study primarily examined the immediate physiological responses to SMCSO, revealing the complexity of determining its overall impact on honey bee health due to the mixed potential benefits and drawbacks. Although SMCSO is predominantly found in rapeseed flowers and other Brassicaceae plants, colony variation studies indicate that SMCSO consumption can vary in nature. Therefore, the study is limited by the scope of natural exposure to SMCSO and its real effects in a natural setting. Furthermore, SMCSO-administered bees showed alterations in amino acid profiles in the gut, yet it remains unclear how the honey bee gut microbiome degrades SMCSO and produces other non-proteinogenic amino acids, such as GABA and ornithine. Consequently, the long-term effects of SMCSO on bee health, as well as its interactions within the broader spectrum of their diet, remain largely unexplored and warrant future research.

## 4. Conclusion

Our study examined the functions of SMCSO, a plant-based non-proteinogenic amino acid that is highly abundant in summer bees. SMCSO and its metabolites 3Me-TLA SO and NAc-SMCSO vary in concentration among honey bee colonies, reflecting diverse foraging behaviours. Concentrations of these three metabolites peaked in midsummer, likely tied to increased foraging, colony development and rapeseed blooming, as evidenced by the presence of SMCSO in rapeseed flower nectar and pollen and its accumulation in rapeseed honey. Foragers showed higher abdominal concentrations of these metabolites than nurse bees. While SMCSO administration did not show toxic effects on bee survival, long-term exposure led to reduced bee body weight, particularly in the head and thorax, suggesting negative impacts on honey bee health. Additionally, it altered amino acid levels in the bee gut, indicating an influence on protein metabolism and digestion. On a positive note, SMCSO exhibited antioxidant properties that showed honey bee resilience against oxidative stress. This comprehensive analysis of SMCSO in honey bee metabolism highlights its significant role in bee physiology, nutrition, growth and seasonal behaviours, emphasizing its implications for pollination services.

## 5. Material and methods

### 5.1. The general procedure for honey bee sampling

All the samples were collected from apiaries in the Czech Republic: Kyvalka (GPS location: 49.1913056 N, 16.4495556 E), Mlada Boleslav (GPS location: 50.4186111 N, 15.0357778 E), Praha-vychod (GPS location: 50.2068611 N, 14.3759444 E), Praha-Suchdol (GPS location: 50.1304167 N, 14.3736111 E) and Bee Research Institute (GPS location: 50.2039797 N, 14.3667811 E) (electronic supplementary material, figure S5).

The beekeepers followed the country’s professional standards. The bees were kept in Langstroth-type hives. Under the climatic and biological conditions in the Czech Republic, honey bees begin brooding in early January, with the most rapid development occurring in May, two to three honey harvests in June and July, and subsequent feeding with sucrose solution in July and August to prepare the colonies for the upcoming winter. The predominant sources of nectar in most of the sampling locations were rapeseed and linden. The selected colonies did not show any clinical symptoms of disease or varroa infestation at sampling time.

Collected honey bee samples (*Apis mellifera carnica*) were dissected using the following method: venom sacs were removed first, and digestive tracts were dissected with sterilized tweezers. Sterilized scalpels were used to cut bodies; the heads and digestive tracts were discarded unless otherwise required for the analysis.

### 5.2. Sample collection and process for NMR analysis

#### 5.2.1. Isolation and identification of the unknown metabolite from honey bees

Honey bee samples were collected in June 2022 from the Bee Research Institute. Eighty honey bees were homogenized by using a laboratory mortar and pestle under liquid nitrogen, resulting in a pooled sample. This sample was subsequently defatted with 20 ml of hexane and chloroform (VWR, Radnor, PA, USA) in a 50 ml tube. The samples were centrifuged at 17 000×*g* for 10 min. The pellet was then dried in a dry bath NDK200-2 (MIULab, Hangzhou, China) under a nitrogen stream and extracted with 25 ml of methanol (MeOH) (Honeywell, Wabash, IN, USA). This extract was dried in a centrifugal vacuum evaporator (MiVac Duo, Genevac, Ipswich, UK) and reconstituted in approximately 40 ml of ultra-purified H_2_O.

The aqueous solution was fractionated on J.T. Baker BAKERBOND™ solid phase extraction (SPE) Aromatic Sulfonic Acid cartridges (3 ml, 500 mg, Avantor, PA, USA). The cartridges were conditioned with 3 ml of MeOH, 3 ml of H_2_O and 3 ml of H_2_O acidified with HCl. After loading, each cartridge was washed with 3 ml of 0.1 M aqueous HCl (VWR, Radnor, PA, USA) and 3 ml of 0.1 M HCl in MeOH. The elution was done with 4 ml of MeOH containing 5% NH_4_OH (VWR, Radnor, PA, USA). The eluate from multiple cartridges was evaporated under a nitrogen stream, then dissolved in ultra-purified H_2_O, and the SMCSO compound was analysed and confirmed by LC-MS/MS and ^1^H NMR.

Initial identification of the compound was done based on a ^1^H STOCSY NMR analysis (electronic supplementary material, figure S1), and the presence of SMCSO was confirmed through spiking with the purchased standard (abcr GmbH, Karlsruhe, Germany) in both ^1^H NMR analysis (electronic supplementary material, figure S2) and in LC-MS/MS (electronic supplementary material, figure S3). Detailed information on the LC-MS/MS is mentioned in electronic supplementary material, table S8. The additional two methyl signals (δ2.75 and δ2.78) present in the NMR spectra near the compound of interest were later identified based on their chemical shift as 3Me-TLA SO and NAc-SMCSO, according to previous research [32,33,39]. These compounds have been discussed in the SMCSO as they are known catabolites in humans [32,33,39].

#### 5.2.2. Experiment 1: variation of SMCSO and its derivatives among the bee colonies and the correlation of their concentrations between the body and the gut

A sample set from a previously published study [30] was utilized to comprehend the variability of SMCSO and the variation of its metabolites among the different bee colonies and the correlations between gut and body. The samples were collected on 27 June 2017, from three randomly selected colonies in the Kyvalka apiary. A total of 29 honey bee samples, 7−13 from each colony, were used for body and gut analysis. The bodies and guts were dissected and collected separately. The sample process, extraction protocol and NMR analysis were described in prior research [30].

#### 5.2.3. Experiment 2: monthly changes of SMCSO and its metabolites

Honey bee samples were collected monthly from March 2018 to February 2019. Apiaries from Mlada Boleslav, Praha-Vychod and Praha-Suchdol provided samples. The samples were collected from two different colonies at each location, apart from December to February, when samples were only collected from two locations (Mlada Boleslav and Praha-Suchdol). Bees were randomly collected from brood combs and placed in cages for 24 h on a 60% (w/v) sucrose solution to remove the bias of short-term dietary influences. Upon collection, the samples were stored at −80°C until the time of the analysis.

From each colony, five honey bees, excluding heads, venom sacs and guts, were selected at random and combined into a single sample, which was homogenized in 5 ml of MeOH (Honeywell, Wabash, IN, USA) using a T 18 digital ULTRA-TURRAX (IKA, Germany). After that, the sample was extracted in an ultrasonic bath for 5 min, centrifuged at 17 000×*g* for 10 min at 4°C, and the resulting supernatant was evaporated in a dry bath under a nitrogen stream as mentioned above. After evaporation, the sample was resuspended in 3 ml of H_2_O, vortexed and centrifuged at 13 000×*g* for 5 min. Subsequently, 540 µl of supernatant was collected and mixed with 60 µl of NMR phosphate buffer (1.5 M K_2_HPO_4_/NaH_2_PO_4_ pH 7.4 in D_2_O, 5 mM 3-(trimethylsilyl)-2,2,3,3-tetradeuteropropionic acid (TSP) and 0.2% NaN_3_; hereafter, it is referred to as the NMR solution) and transferred into NMR tubes (5 mm, 7″, High-Throughput, Willmad, NJ, USA) for analysis. The same tubes were used for all NMR analyses.

#### 5.2.4. Experiment 3: metabolite examination in each body part of worker bees (head, thorax, abdomen, gut and haemolymph)

Honey bee workers were collected on 8 July 2020, from a single colony located at the Bee Research Institute. Nurse bees from brood combs with hairy thoraxes and foragers returning to the hive entrance with hairless thoraxes and pollen on hind legs were collected and transported to the laboratory. Bees were anaesthetized with CO_2_ for 10 s, separated into single body parts (head, thorax, abdomen and gut), and pooled five individuals per sample into 2 ml microtubes. Approximately 20 μl of haemolymph was drawn from the bees using glass capillary tubes, which were injected in the intersegmental space between abdominal terga 2 and 3, and then the abdomen was gently pressed. The capillaries were then emptied by air pressure, and multiple collections were pooled in a 0.2 ml microtube. The collected samples were placed immediately into a dry ice box and stored at −80°C until the metabolite extraction.

Each body part of the pooled sample, except for the gut and haemolymph, was homogenized using a laboratory mortar and pestle under liquid nitrogen. The gut was homogenized using a disposable pestle, while methanol was contained in the tube.

The homogenized samples were extracted with methanol using a previously described protocol [76]. For NMR analysis, the dried tissue samples were resuspended in 600 µl of D_2_O, vortexed, and centrifuged at 12 000×*g* for 5 min. Subsequently, 540 µl of the supernatant was collected, mixed with 60 µl of NMR solution and transferred into NMR tubes. The haemolymph for NMR analysis was dissolved in 600 µl of D_2_O, vortexed, and centrifuged at 12 000×*g* for 5 min. The supernatant in the volume of 540 µl was mixed with the 60 µl of NMR solution and transferred into NMR tubes.

#### 5.2.5. Experiment 4: experimental oral exposure of honey bees to SMCSO

To evaluate its biological effect, he SMCSO (abcr GmbH, Karlsruhe, Germany) was administered to the newly emerged honey bee workers. The honey bees were collected in the middle of October 2022 from six brood frames with capped brood that were isolated from four non-sister colonies at the apiary in Kyvalka. The frames were transported to the laboratory, where they were kept in the dark at 34 ± 1°C and 80% relative humidity for one day to let the new bees emerge. Approximately 550 bees were collected and further kept in plastic cages [76]. Specifically, four groups per 100 bees were used to test the effect of SMCSO acute feeding (AC), and two groups per 50 bees were determined to test the long-term (LT) feeding effect.

In the AC experiment, a bee group (100 bees) were fed with 1 mg of SMCSO dissolved in 1 ml of sucrose solution (1:1 w/v; sucrose/water), which was replaced by sucrose solution when the SMCSO dose was completely fed, i.e. 3 days after the administration. Another group (100 bees) were used as controls and fed sucrose solution without SMCSO from the start of the experiment.

In the LT experiment, one group of 50 bees was provided with SMCSO (1 mg ml^−1^) dissolved in sucrose solution (1:1 w/v; sucrose/water) ad libitum during the whole course of the experiment, and the control group (50 bees) was fed with sucrose solution without SMCSO.

All honey bee groups were provided with sterile pollen ad libitum, and their survival was scored daily for 10 days. The haemolymph was sampled from 10-day-old honey bees by cutting off the abdomen, gently pressing the thorax and collecting 2 µl of haemolymph per bee into PBS with phenylthiourea (1 mg ml^−1^; haemolymph dilution 1.25 times). Ten honey bees were pooled in each haemolymph sample and stored at −80°C until further use [77]. The tissue homogenates were prepared by freezing the intact 10-day-old honey bees at −80°C immediately after their collection and further processing, according to previous research [76]. Briefly, each honey bee was weighed, dissected into body parts (head, thorax, abdomen and gastrointestinal tract with sting and venom sac), and homogenized on ice using a pestle. For the homogenization, 10 µl of PBS (pH 7.4) was added per milligram of tissue.

For the NMR analysis, five bees were randomly selected and dissected for each group. The bodies (excluding the venom sac and head) and guts were extracted in methanol and processed in the same manner as described in §5.2.4.

#### 5.2.6. Experiment 5: SMCSO confirmation in nectar and pollen of rapeseed flowers

On 23 April 2024, at 16.00, during cloudy weather, five rapeseed varieties were collected at the Czech University of Life Sciences Prague, research station Cerveny Ujezd (GPS location: 50.0727150 N, 14.1714506 E). The varieties sampled included Exbury (EX), Chetta (CH), PX 131, ES Capelo (CAP) and Salute (SAL). The parcels were designated as follows: OPT 10/B DK Exbury, OPT 8/B Chetta, OPT 1/B PX 131(1), OPT 6/B ES Capelo and OPT 2/B Salute. From each crop, 40 open flowers were collected in duplicates using 50 ml tubes and transferred to the laboratory immediately. Upon arrival at the laboratory, 5 ml of ultra-purified water was added to each tube, which was then gently shaken for 5 s. The water was transferred to a new 15 ml tube. This washing step was repeated three times, pooling the extracts to obtain approximately 12 ml of liquid containing pollen and nectar. The pooled extracts were centrifuged at 2100×*g* for 5 min. The resulting pellet (pollen) was separated from the supernatant. Both the pellet and supernatant samples were frozen at −80°C for further analysis.

Supernatant samples containing nectar were placed in a freeze dryer overnight, dried residue was reconstituted with 540 µl of ultra-purified water, mixed with 60 µl of NMR solution, and transferred into NMR tubes. For pollen samples, the pellets were extracted with 2 ml of methanol, ultrasonicated for 5 min, and centrifuged at 2100×*g* for 10 min. The supernatant was collected and dried under a centrifugal vacuum concentrator at 40°C (MiVac Duo, Genevac, Ipswich, UK). The dried residue was reconstituted with 540 µl of ultra-purified water, mixed with 60 µl of the same NMR solution used for the nectar samples, and transferred into NMR tubes. The presence of SMCSO was confirmed by spiking the samples with the purchased standard (abcr GmbH, Karlsruhe, Germany).

#### 5.2.7. Experiment 6: SMCSO confirmation in honeys

A total of 30 honey samples, representing three different types (rapeseed, black locust and honeydew), were obtained from the Bee Research Institute (Maslovice, Czech Republic). All samples were collected in 2019 and analysed in August of the same year. Honey samples were collected in 15 ml tubes using a metal spatula and stored in the dark at room temperature. The samples were identified by professional beekeepers based on their appearance and sensory characteristics. Prior to analysis, the samples were placed in a thermostat at 45°C for 1 h to dissolve any crystals formed. For each sample, 240 ± 10 mg of honey was weighed into a microtube and 830 μl of ultra-purified water was added to achieve a final concentration of approximately 240 mg ml^−1^. The solution was vortexed and subsequently placed in an ultrasonic bath for 5 min. Following this preparation, 540 μl of the solution was pipetted into a clean microtube and mixed with 60 μl of NMR solution. The final mixture was transferred into 5 mm NMR tubes for analysis.

### 5.3. Determination of toxicity, antioxidant capacity and antimicrobial activity in honey bees

The total antioxidant capacity was determined in honey bee haemolymph and tissue homogenates using the oxygen radical absorption capacity assay according to a published protocol [77]. Specifically, 10 μl of samples (haemolymph was prepared as described above; tissue homogenates were diluted 200 times in PBS, pH 7.4) and serial dilutions of standard antioxidant 6-hydroxy-2,5,7,8-tetramethylchromane-2-carboxylic acid (Trolox; Sigma-Aldrich, St Louis, MO, USA) were mixed in a black microtitration plate (Brand, Wertheim, Germany) with 170 μl of 100 nM fluorescein sodium salt (Sigma-Aldrich, St Louis, MO, USA) and incubated for 10 min in the dark at 37°C. To produce oxygen radicals, 20 μl of ice-cold 0.1 M 2,20-azobis(2-amidinopropane) dihydrochloride (Sigma-Aldrich, St Louis, MO, USA) was added, and the fluorescence (excitation 460 nm/emission 535 nm) was measured every 30 s for 2 h at 37°C with shaking before the first read using a Sense plate reader (Hidex, Turku, Finland). The antioxidant capacity of samples was determined as the Trolox concentration with equivalent antioxidant activity.

The antimicrobial activity in haemolymph samples was determined using the radial diffusion assay as described previously [77]. Briefly, the antimicrobial effect was measured against the Gram-positive bacterium *Micrococcus luteus* (CCM169) dispersed in 4% LB agar (MO BIO Laboratories, Inc., Carlsbad, CA, USA). The samples and lysozyme standards (EC 3.2.1.17; Sigma-Aldrich, St Louis, MO, USA) were loaded in a volume of 5 μl into the wells prepared in the agar and incubated for 24 h at 30°C until clear inhibition zones appeared around the wells. The diameter of zones was measured, and the antimicrobial activity of samples was calculated as the concentration of lysozyme with equivalent bacterial growth-inhibiting activity.

### 5.4. Data processing and statistical analysis

NMR analysis and data processing. The 1D ^1^H NMR spectra were acquired using the 1D NOESY Bruker pulse program in a Bruker Avance III spectrometer (Bruker, Billerica, MA, USA) operating at 500.23 MHz for ^1^H observation at 298 K, equipped with a 5 mm broadband observation probe. All spectra were acquired at 4 s acquisition time, 1 s relaxation delay (D1), 0.1 s mixing time, spectral width of 8012.82 Hz, 64 k data points and 128 scans (64 scans were used for the dataset from 2017 reporting the variation among colonies). All spectra were zero-filled to 128 k, exponentially multiplied (line broadening of 0.3 Hz), and manually phased using Topspin 3.5 (Bruker, Billerica, MA, USA).

The spectra were pre-processed with an in-house script described in previous research [78]. The baseline was corrected by a multipoint baseline correction in user-defined segments. Spectra between 0.5 and 9.5 ppm, excluding the residual water region (4.90−4.75 ppm) and the methanol region (3.75−3.34 ppm), were reduced into defined buckets. The intervals for the bins were determined after annotating a subset of spectra in the Chenomx NMR Suite ver. 9.0 (Chenomx, Edmonton, AB, Canada), comprising the built-in reference library and our in-house database. For quantitative NMR of SMCSO and its derivatives, peak integrals were quantified by a ratio analysis normalized to the number of protons [79] and finally normalized to 1 g of the tissue. The haemolymph metabolite concentrations were normalized to proline and expressed as a molar ratio to proline. SMCSO in pollen and nectar was normalized by scaling up to 100 fresh flowers, while in honey it was normalized to 1 g of honey.

If not stated otherwise, the statistical analysis and the data visualization were performed in R Studio version 4.1.2 (Rstudio, Boston, MA, USA). Packages of dplyr version 1.1.4 [80] and rstatix version 0.7.2 [81] were used for data shaping and statistical analysis. The data from the metabolite comparisons among colonies, the seasonal comparison, and honey comparison study were analysed using the Kruskal–Wallis test followed by Dunn’s procedure, with *p*-values adjusted using the Benjamini–Hochberg procedure. The Wilcoxon rank-sum test and the Benjamini–Hochberg procedure for false discovery rate correction were applied to compare the foragers and nurses on each body part. Spearman’s rank-order correlation was performed to compare and assess the relationship between the metabolite concentration in honey bee guts and bodies. Data visualization was realized by using gridExtra version 2.3 [82], ggpmisc version 0.6.0 [83], ggpubr 0.6.0 [84] and ggplot2 version 3.4.2 [85]. Statistical analysis of data from SMCSO feeding experiments was performed in Prism 9.0 (GraphPad Software, San Diego, CA, USA). The survival curves were compared using the Mantel–Cox test, and the unpaired *t*‐test was used to compare differences between the control and SMCSO-exposed groups. Data from NMR analysis of honey bee bodies and guts from SMCSO feeding experiments were plotted using MetaboAnalyst 5.0 [86]. Data were processed using probabilistic quotient normalization, log transformation and auto-scaling. PCA and OPLS-DA with permutation test validation for 100 applications and VIP were applied. The OPLS-DA models were evaluated according to good fit (R^2^Y) and model predictive ability (Q^2^). Results with *p*-values less than 0.05 were considered statistically significant.

## Data Availability

Supplementary material, data and the R codes are available online [87].
